# CASCADE: Context-Aware Data-Driven AI for Streamlined Multidisciplinary Tumor Board Recommendations in Oncology

**DOI:** 10.3390/cancers16111975

**Published:** 2024-05-23

**Authors:** Dania Daye, Regina Parker, Satvik Tripathi, Meredith Cox, Sebastian Brito Orama, Leonardo Valentin, Christopher P. Bridge, Raul N. Uppot

**Affiliations:** 1 Massachusetts General Hospital, Boston, MA 02114, USA; stripathi3@mgh.harvard.edu (S.T.); meredith.cox@duke.edu (M.C.); leonardo.valentin@mgh.harvard.edu (L.V.); cbridge@mgh.harvard.edu (C.P.B.); uppot.raul@mgh.harvard.edu (R.N.U.); 2 Harvard Medical School, Boston, MA 02115, USA; reginafrancesparker@gmail.com; 3 Athinoula A. Martinos Center for Biomedical Imaging, Charlestown, MA 02129, USA; 4 Baylor College of Medicine, Houston, TX 77030, USA; sebastian.brito-orama@bcm.edu; 5 Professional Hospital Guaynabo, Guaynabo 00971, Puerto Rico

**Keywords:** machine learning, computer algorithm, hepatocellular carcinoma, multidisciplinary decision-making, tumor board

## Abstract

**Simple Summary:**

This research aims to evaluate the effectiveness of a machine learning algorithm, XGBoost, in predicting treatment recommendations for patients with hepatocellular carcinoma (HCC). The study uses clinical and imaging data from patients discussed at a multidisciplinary tumor board. The findings suggest that the algorithm can accurately predict all eight treatment recommendations made by the board, potentially aiding clinical decision-making in settings lacking subspecialty expertise.

**Abstract:**

This study addresses the potential of machine learning in predicting treatment recommendations for patients with hepatocellular carcinoma (HCC). Using an IRB-approved retrospective study of patients discussed at a multidisciplinary tumor board, clinical and imaging variables were extracted and used in a gradient-boosting machine learning algorithm, XGBoost. The algorithm’s performance was assessed using confusion matrix metrics and the area under the Receiver Operating Characteristics (ROC) curve. The study included 140 patients (mean age 67.7 ± 8.9 years), and the algorithm was found to be predictive of all eight treatment recommendations made by the board. The model’s predictions were more accurate than those based on published therapeutic guidelines by ESMO and NCCN. The study concludes that a machine learning model incorporating clinical and imaging variables can predict treatment recommendations made by an expert multidisciplinary tumor board, potentially aiding clinical decision-making in settings lacking subspecialty expertise.

## 1. Introduction

Management of hepatocellular carcinoma (HCC) is complex due to the highly variable pathology of the cancer as well as its frequent coexistence with other complex medical issues, such as cirrhosis and viral hepatitis infection [1,2,3]. According to the American Association for the Study of Liver Diseases (AASLD), the treatment of HCC involves a broad spectrum of clinical practice, including surveillance of patients with cirrhosis for HCC, establishing the diagnosis of HCC, and various therapeutic options for the treatment of HCC [4,5]. In the early stages, when a tumor is small, HCC treatment may include surgery, transplant, and ablation. Conversely, after the cancer grows and spreads, a combination of immunotherapy and targeted therapy may be the gold standard treatment [6,7,8]. These treatments include surgical tumor resection, liver transplantation, locoregional treatments such as ablation, transarterial chemoembolization (TACE), selective internal radiotherapy (SIRT) with yttrium-90 (Y-90), systemic chemotherapy, and external beam radiotherapy. Numerous factors drive the selection of treatment. These include the tumor staging, number of tumors, location of tumors, vascular involvement, serum biomarkers (bilirubin, albumin, prothrombin time, sodium, creatinine), goals of treatment (palliative or curative), relative contraindications or patient comorbidities, candidacy for transplantation or clinical trials, patient preferences, physician preferences, and available resources [4,9].

For these reasons, multidisciplinary team meetings (tumor boards) are considered best practice in the management of patients diagnosed with HCC. Tumor boards typically consist of oncologists, surgeons, diagnostic and interventional radiologists, radiation oncologists, pathologists, and other healthcare professionals [7]. The combined input of individuals with specialized expertise helps to optimize clinical decision-making. Despite the collective expertise of tumor boards, decision-making can sometimes be flawed. Specifically, decisions may be hindered by weaknesses in internal team processes, limitations in the quantity and quality of information available to all board members, and group or individual biases, resulting in variability in the final treatment plan [10,11]. Furthermore, these resource-intensive tumor boards are not readily accessible to all 750,000 patients diagnosed annually with HCC and tend to be restricted to a few medical centers [12].

Considering these limitations, there is interest in the role of artificial intelligence algorithms in mimicking or supplementing the multidisciplinary decision-making of tumor boards. Several studies have demonstrated the utility of artificial intelligence to predict both diagnostic and clinical decision-making [13,14,15]. However, limited published data have demonstrated the utility of machine learning in predicting multidisciplinary decision-making [16]. The purpose of this paper is to explore the role of machine learning in predicting treatment recommendations of a multidisciplinary HCC tumor board at a quaternary academic medical center. 

## 2. Materials and Methods

### 2.1. Patient Population

In this retrospective study, patients were enrolled if they met the following inclusion criteria: (1) age of 18 years old or above; (2) diagnosed with HCC and not any other type of cancer; (3) referred to oncology at Massachusetts General Hospital (MGH); and (4) discussed at the multidisciplinary tumor board at MGH between August 2017 and August 2019. A total of 140 patients met these criteria and were included in the study. The study obtained Institutional Review Board (IRB) approval from the institutional IRB, and the need for informed consent was waived.

### 2.2. Tumor Board Composition

The tumor board met on a weekly basis for 1 h sessions throughout the 2-year period from August 2017 to August 2019. Each tumor board meeting consisted of consistent medical oncologist, a surgical oncologist, a transplant surgeon, a radiation oncologist, and an interventional radiologist. 

The recommendations of the tumor board were categorized into 8 options—(1) intra-arterial therapies, including conventional transarterial chemoembolization (TACE), drug-eluting bead TACE (DEB-TACE), (2) selective internal radiation therapy (SIRT); (3) percutaneous ablation, with microwave ablation (MWA) (4) radiotherapy, including external beam radiotherapy (EBRT) or stereotactic body radiotherapy (SBRT); (5) surgical resection; (6) transplant; (7) chemotherapy; and (8) palliative.

The board recommended at least one treatment option, and potentially multiple treatment options, to each patient. The aim of this study was to develop a computer algorithm modeling these initial recommendations of the tumor board. 

### 2.3. Machine Learning Algorithm

For each patient, a total of 24 clinical and imaging variables were extracted from the medical record to be included in the machine learning algorithm; patient characteristic values are shown in Table 1. These variables were the same features considered by the tumor board in making treatment recommendations and were provided by the tumor board. All the features provided by the tumor board were included in the model with no exclusions. These variables included patient demographics, number of enhancing liver tumors, tumor size, tumor location, number of Organ Procurement and Transplantation Network (OPTN) tumors, Model for End-Stage Liver Disease (MELD) score, alpha-fetoprotein (AFP), and total serum bilirubin. For each recommended treatment plan, the treated patient cohort was split between a training and testing set at a ratio of 4:1 while keeping the ratio of those recommended and not recommended for a treatment the same for the training and testing set. For the respective training and testing sets, missing variables were imputed using a k-nearest neighbor algorithm. Leave-one-out cross-validation was used in the test sets. The XGBoost machine learning algorithm was used to create a separate classifier for each treatment plan. This model was used after comparing the performance of several different types of algorithms, including XGBoost, random forest, regression, support vector machine, and decision tree algorithms. XGBoost with a depth of 5 and learning rate of 0.001 led to the best results. Model performance was assessed using AUC-ROC analysis and accuracy.

## 3. Results

### 3.1. Patient Population

A total of 140 patients were enrolled in the study (Table 1). The mean patient age was 67.7 ± 8.9 years. A total of 110 patients (79%) were male; 7 patients (5%) had extrahepatic disease. The mean MELD score was 14.5 ± 5.81. The mean AFP was 22,996.7 ± 130,277.3 ng/mL. The mean total bilirubin was 1.8 ± 2.1 mg/dL. There were 205 total tumors among the study participants. A total of 82 tumors (40%) were in the left hepatic lobe, and 123 tumors (60%) were in the right hepatic lobe. A total of 156 tumors (76%) were OPTN 5 tumors. The mean tumor size was 4.71 ± 3.6 cm. 

### 3.2. Tumor Board Recommendations

The tumor board recommended at least one of the eight different treatment options to each patient. The eight treatment options included intra-arterial therapies, radioembolization, ablation, radiotherapy, surgical resection, transplant, palliative, chemotherapy, and hepatic transplantation. These options included intra-arterial therapy (TACE/DEB-TACE) for 30.00% of patients, SIRT/Radioembolization for 18.57%, ablation (MWA) for 37.86%, radiotherapy (EBRT/SBRT) for 26.43%, surgical resection for 15.71%, transplant for 12.86%, chemotherapy for 10.17%, and palliative treatment for 7.14% of patients (Table 2).

### 3.3. Model Performance

**Figure 1:**
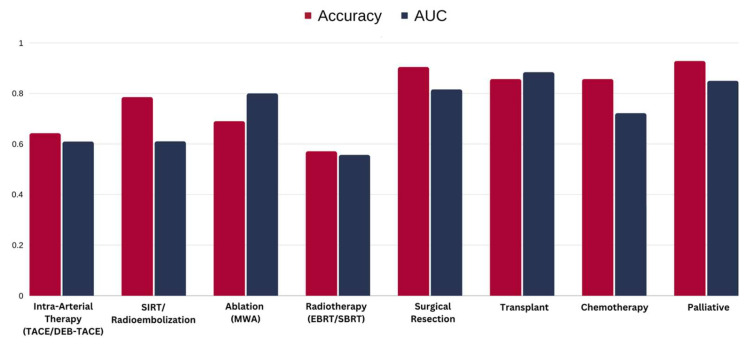
Machine learning model performance for each treatment option. TACE: transarterial chemoembolization, DEB-TACE: drug-eluting bead transarterial chemoembolization, SIRT: selective internal radiation therapy, MWA: microwave ablation, EBRT: external beam radiotherapy, SBRT: stereotactic body radiotherapy.

The XGBoost classifiers using the clinical variables were predictive of all eight treatment recommendations made by the tumor board, although performance varied significantly between the treatments. The performance of the machine learning model was assessed using the area under the ROC Curve (AUC), shown in Table 2. The ROC curve plots the true positive rate against the false positive rate and, thereby, reflects the tradeoff between sensitivity and specificity. A higher AUC corresponds to a more accurate prediction by the model [17,18]. These categories encompassed a range of therapeutic approaches, including intra-arterial therapy (TACE/DEB-TACE) with an accuracy of 0.64 and an AUC of 0.61, SIRT/radioembolization with an accuracy of 0.78 and an AUC of 0.61, ablation (MWA) with an accuracy of 0.69 and an AUC of 0.80, radiotherapy (EBRT/SBRT) with an accuracy of 0.57 and an AUC of 0.55, surgical resection with an accuracy of 0.90 and an AUC of 0.81, transplant treatments with an accuracy of 0.85 and an AUC of 0.88, chemotherapy with an accuracy of 0.85 and an AUC of 0.72, and palliative care with an accuracy of 0.92 and an AUC of 0.85, respectively (Figure 1). 

## 4. Discussion

This study demonstrates that a machine learning model can be predictive of the treatment recommendations of a multidisciplinary HCC tumor board at an academic medical center. The AUCs for the majority of the treatment options—ablation, chemotherapy, surgical resection, transplant, and palliative—were over 72%. 

Initial experience in developing a computer algorithm to predict multidisciplinary decisions in HCC and transplant patients was reported by Valentin et al. [19]. Beyond this work, there is one additional study with the aim of developing a computer algorithm to predict multidisciplinary decision-making that may be used as a point of comparison for our model. A study by Lin et al. developed a machine learning model able to predict tumor board decisions about adjuvant systemic therapy in early breast cancer [16]. This model considered a smaller number of treatment options than did our model. The AUC for the treatment options considered in this model ranged from 0.78 to 0.99, and the predictions of this model were shown to be more accurate than those based on published therapeutic guidelines by ESMO and NCCN [16]. As the AUCs for the majority of treatment options considered in our model were in a similar range, our machine learning algorithm was comparable in terms of predictive potential to this model developed by Lin et al. for breast cancer. 

The findings of the present study did demonstrate low accuracy in predicting TACE/SIRT and chemotherapy treatment recommendations. Interestingly, the model developed by Lin et al. also reported a lower accuracy in predicting the chemotherapy treatment recommendation. The study did not comment on TACE or SIRT, as these modalities do not play a role in breast cancer treatment. Lin et al. hypothesized that chemotherapy-specific decision variations may have arisen in part due to divergences in resource availability. Variations in resource availability may have similarly contributed to our model’s relatively lower performance in predicting chemotherapy and also TACE/SIRT-specific decisions. Additionally, the clinical variability in recommending TACE/SIRT or chemotherapy, given that TACE/SIRT is a relatively new and evolving therapy and given the ever-changing landscape of chemotherapy options, may have contributed to our model’s lower performance in predicting these treatment recommendations.

There are numerous potential applications of this model in the future. For example, this model could be used to support existing multidisciplinary decision-making at academic medical centers, both small and large [20,21,22,23,24]. Furthermore, this model could be used to spread multidisciplinary expertise to more remote areas where such expertise may not be available, thereby benefiting a greater number of patients [23]. 

The utility of ML models in predicting treatment recommendations extends beyond HCC to other oncologic areas as well. For instance, in breast cancer, machine learning models have been used to predict the likelihood of recurrence and to guide the selection of adjuvant therapy. These models take into account various factors such as tumor size, grade, hormone receptor status, and genomic markers to make their predictions [25,26,27,28,29].

In lung cancer, machine learning models have been developed to predict the response to immunotherapy. These models use data from imaging studies, clinical parameters, and molecular markers to predict which patients are likely to respond to treatment [30,31,32,33,34,35].

In colorectal cancer, machine learning models have been used to predict the risk of metastasis and to guide the use of adjuvant chemotherapy. These models consider factors such as tumor stage, lymph node involvement, and molecular markers [36,37,38,39].

In each of these cases, the ML model serves as a decision support tool, helping the multidisciplinary tumor board to make evidence-based treatment recommendations. However, it is important to note that these models are not meant to replace the clinical judgment of the tumor board but rather to augment it. The final treatment decision should always be made in the context of a thorough discussion among the multidisciplinary team, taking into account the patient’s preferences and overall health status. It is also worth noting that while these models show promise, they are still in the early stages of development and validation. Further research is needed to refine these models and to evaluate their impact on patient outcomes in the real-world setting. 

There are several limitations of this study. First, this is a retrospective study with a relatively small cohort; as a consequence, the strength of the results may not be generalizable. It will be helpful to validate the accuracy of this machine learning model with a larger number of patients in predicting tumor board decisions prospectively. While the algorithm used in the present study was able to predict decisions retrospectively, the development of a prospective model may be more helpful but comes with unique challenges [40], such as the risk of overfitting and complications arising from the dynamic nature of medicine [41,42,43]. Second, it will be useful to assess the model’s efficacy at academic medical centers outside our institution to account for potential institutional biases and to ensure model generalizability [44,45]. Third, expanding the study to include a larger number of patients will facilitate the generalizability and interpretation of our results. Finally, it will be important to consider the compatibility of the model with human cognitive processes in order to validate the safety of a model for use in clinical settings. A machine learning algorithm that has achieved a high degree of accuracy is not necessarily acceptable for use in clinical settings, as issues may arise relating to human/computer interaction that threaten the efficacy of the model [46]. 

## 5. Conclusions

This study demonstrated the utility of a machine learning model in predicting the treatment recommendations of a multidisciplinary HCC tumor board at an academic medical center. Future applications of this model range from supporting existing decision-making at large academic medical centers to spreading multidisciplinary expertise to more remote areas. As we work toward these goals, further research will be required to validate the model prospectively, evaluate the model more broadly, and continuously improve and assess the model’s compatibility in a clinical context.

## Figures and Tables

**Table 1:** Patient characteristics.

Total Study Participants (*n*)	140 Patients
Gender	Male—110 patients (79%)
	Female—30 patients (21%)
Age years	67.7 ± 8.9
Patients with extrahepatic disease *n*	7 (5%)
MELD score	14.5 ± 5.81
AFP ng/mL	22,996.7 ± 130,814.5
Total bilirubin mg/dL	1.8 ± 2.1
Total number of tumors *n*	205
Location of tumors per hepatic lobe *n* (%)	Left hepatic lobe—82 (40%)
	Right hepatic lobe—123 (60%)
Location of tumors per hepatic segment *n* (%)	Segment 1—1 (0.5%)
	Segment 2—11 (5.4%)
	Segment 3—11 (5.4%)
	Segment 4a—19 (9.3%)
	Segment 4b—16 (7.8%)
	Segment 5—28 (13.7%)
	Segment 6—32 (15.6%)
	Segment 7—42 (20.5%)
	Segment 8—45 (21.9%)
OPTN 5 tumors *n* (%)	156 (76%)
Mean tumor size cm	4.71 ± 3.6

MELD: Model For End-Stage Liver Disease; AFP: Alpha Fetoprotein; OPTN 5: Organ Procurement and Transplantation Network classification 5.

**Table 2:** Tumor board recommendations *.

Treatment Recommendation	Number of Patients *n* (%)
Intra-arterial therapy (TACE/DEB-TACE)	42 (30.00%)
SIRT/Radioembolization	26 (18.57%)
Ablation (MWA)	53 (37.86%)
Radiotherapy (EBRT/SBRT)	37 (26.43%)
Surgical resection	22 (15.71%)
Transplant	18 (12.86%)
Chemotherapy	15 (10.71%)
Palliative	10 (7.14%)

* Note that patients may have been recommended more than one treatment option.

## Data Availability

Data are contained within the article text.
